# The Non-Relativistic Limit for the e-MHD Equations

**DOI:** 10.1155/2014/261082

**Published:** 2014-01-30

**Authors:** Hongli Wang, Jie Zhao

**Affiliations:** ^1^College of Mathematics and Information Science, North China University of Water Resources and Electric Power, Zhengzhou 450011, China; ^2^Zhengzhou Huimin Middle School, Zhengzhou 450000, China

## Abstract

We investigate the non-relativistic limit for the e-MHD equations in a three-dimension unit periodic torus. With the prepared initial data, our result shows that the small parameter problems have unique solutions existing in the finite time interval where the corresponding limit problems (incompressible Euler equations) have smooth solutions. Moreover, the formal limit is rigorously justified.

## 1. Introduction

Let us consider the following so-called electron-magnetohydrodynamics (e-MHD) system [1, 2]:

(1) $$\begin{array}{c} \partial_{t}u+\left(u\cdot \nabla \right)u+E+\gamma u\times B=0,\\ \partial_{t}B+\frac{1}{\gamma }\nabla \times E=0,\\ u=-\frac{1}{\gamma }\nabla \times B,\;\mathrm{div}B=0, \end{array}$$

for (*x*, *t*) ∈ *Ω* × [0, *T*], where *Ω* = (ℝ/2*π*)^3^ is the 3-dimensional torus. The unknowns are *u* ∈ ℝ^3^, *E* ∈ ℝ^3^, and *B* ∈ ℝ^3^. In the above equations, the non-dimensionless parameter *σ* is proportional to 1/*c*, where *c* = (*ϵ*_0_ *ν*_0_)^−1/2^ is the speed of light, with *ϵ*_0_ and *ν*_0_ being the vacuum permittivity and permeability. Let us notice that, as *γ* → 0 in (1), we get ∇×*E* = 0(⇒*E* = ∇*p* ^*I*^), ∇×*B* = 0, div⁡*B* = 0(⇒*B* = 0), and the incompressible Euler equations of ideal fluid:

(2) $$\begin{array}{c} \partial_{t}u^{I}+\left(u^{I}\cdot \nabla \right)u^{I}+\nabla p^{I}=0,\;\mathrm{div}u^{I}=0. \end{array}$$

The goal of this paper is to justify the above formal derivation of the incompressible Euler equations for periodic initial-value problems (IVPs) with an emphasis on three space dimensions. Indeed, it is well known that the phenomenon of non-relativistic is important in many physical situation involving various non-equilibrium processes. For instance, important examples occur in inviscid radiation hydrodynamics [3], quantum mechanics [4], Klein-Gordon-Maxwell system [5], and so on.

The rigorous derivations of the e-MHD equation (1) from Vlasov-Maxwell system equations by a scaling limit and from Euler-Maxwell system by a quasineutral regime are obtained, respectively, in [2] and in [6]. In [7], the incompressible Euler equation (2) of ideal fluid from the e-MHD system (1) via a non-relativistic limit was gotten only in formal derivation. The aim of this work is to give a rigorous justification of the asymptotic limit using the energy method.

Let us recall that the non-relativistic limit *γ* → 0 or *c* → *∞* has been investigated in a few fields. For instance, the limit *γ* → 0 has been performed in Vlasov-Poisson system in [8], in isentropic relativistic Euler equations [9], in a model system for multiple space dimensions radiation hydrodynamics in [10], and in Euler-Maxwell equations [11, 12].

Now we recall some results on the Moser-type calculus inequalities in Sobolev spaces and the local existence of smooth solutions for symmetrizable hyperbolic equations for later use in this paper.

**Lemma 1 (Moser-type calculus inequalities; see [13, 14])**

Let *s* ≥ 1 be an integer. Suppose *u* ∈ *H* ^*s*^(*𝒯* ^3^), ∇*u* ∈ *L* ^*∞*^(*𝒯* ^3^), and *v* ∈ *H* ^*s*−1^(*𝒯* ^3^)∩*L* ^*∞*^(*𝒯* ^3^). Then for all multi-indexes |*α* | ≤*s*, one has (∂_*x*_^*α*^(*uv*) − *u*∂_*x*_^*α*^ *v*) ∈ *L* ^2^(*𝒯* ^3^) and

(3) ||∂xα(uv)−u∂xαv||  ≤Cs(||∇u||0,∞||D|α|−1v||+||D|α|u||||v||0,∞),

where

(4) $$\begin{array}{c} ||D^{h}u||=\underset{\left|\alpha \right|=h}{\sum }||\partial_{x}^{\alpha }u||,\;\forall h\in \mathbb{N}. \end{array}$$

Moreover, if *s* ≥ 3, then the embedding *H* ^*s*−1^(*𝒯* ^3^)↪*L* ^*∞*^(*𝒯* ^3^) is continuous and one has

(5) $$\begin{array}{c} {||uv||}_{s-1}\leq C_{s}{||u||}_{s-1}{||v||}_{s-1},\\ ||\partial_{x}^{\alpha }\left(uv\right)-u\partial_{x}^{\alpha }v||\leq C_{s}{||u||}_{s}{||v||}_{s-1}. \end{array}$$

Recalling the classical result on the existence of sufficiently regular solutions of the incompressible Euler equations (2), we have the following regularity result about (*u* ^*I*^, *p* ^*I*^).

**Lemma 2 (see [15, 16])**

Let *u*_0_^*I*^ satisfy *u*_0_^*I*^ ∈ *C* ^*∞*^ and div *u*_0_^*I*^ = 0. Then there exist 0 < *T*_∗_ < *∞*, the maximal existence time, and a unique smooth solution (*u* ^*I*^, *p* ^*I*^) of the incompressible Euler equations (2) on [0, *T*_∗_) with initial datum *u*_0_^*I*^ satisfying, for any *T*_0_ < *T*_∗_,

(6) $$\begin{array}{c} \left(u^{I},p^{I}\right)\in C^{\infty }\left(\Omega \times \left[0,T_{0}\right)\right). \end{array}$$

This paper is organized as follows. In Section 2 we give some analytical backgrounds and the main result. The proof of the main result is given in Section 3.

## 2. Main Result

First we will state the existence of smooth local solutions for system (1) for the smooth initial data given by

(7) $$\begin{array}{c} {u|}_{t=0}=u_{0},\;\;{B|}_{t=0}=B_{0}. \end{array}$$

**Proposition 3 (see [2, 17])**

Assume that (*u*_0_, *B*_0_) belongs to *C* ^*∞*^(*Ω*) and satisfies

(8) $$\begin{array}{c} -\nabla \times B_{0}=\gamma u_{0},\;\mathrm{div}B_{0}=0. \end{array}$$

Then there exist 0 < *T* ^*γ*^ < +*∞*, the maximal existence time, which depends only on the initial data, and a unique smooth solution (*u*, *B*, *E*) ∈ *C* ^*∞*^(*Ω* × [0, *T* ^*γ*^)) of the incompressible e-MHD equations (1) defined on [0, *T* ^*γ*^).

For the convergence of the e-MHD system (1), our main result is stated as follows.

**Theorem 4**

Let *s*_0_ ∈ *ℕ* with *s*_0_ > (3/2) + 1. Let *u*_0_^*I*^ ∈ *C* ^*∞*^(*Ω*) satisfy div *u*_0_^*I*^ = 0 and let (*u*_0_, *E*_0_, *B*_0_) ∈ *C* ^*∞*^(*Ω*) satisfy (8). Assume that

(9) $$\begin{array}{c} {||\left(u_{0}-u_{0}^{I},B_{0}\right)||}_{H^{s_{0}}(\Omega )}\leq C\gamma \end{array}$$

for some positive constant *C* independent of *γ*. Let *T*_⋆_ be the maximal existence time of the smooth solution (*u* ^*I*^, *p* ^*I*^) ∈ *C* ^*∞*^(*Ω* × [0, *T*_⋆_)) of the incompressible Euler equations (2). Then, for any *T*_0_ < *T*_⋆_, there exist constants *γ*_0_(*T*_0_) > 0 and *C*(*T*_0_) > 0, depending only on *T*_0_ and the initial data, such that the e-MHD system (1) has a classical smooth solution (*u*, *E*, *B*), defined on [0, *T*_0_], satisfying

(10) $$\begin{array}{c} {||\left(u-u^{I},B-\gamma b^{I}\right)||}_{H^{s_{0}}(\Omega )}\leq C\gamma \end{array}$$

for all 0 < *γ* ≤ *γ*_0_ and 0 < *t* ≤ *T*_0_, where the function *b* ^*I*^ satisfies −∇×*b* ^*I*^ = *u* ^*I*^ and div *b* ^*I*^ = 0.

## 3. Proof of Theorem 4

Now we begin to justify the convergence of e-MHD equations to incompressible Euler equations when *γ* → 0. To this end, by the local existence theory and extension method, it suffices to obtain the uniform estimates of the smooth solutions to (1) with respect to the parameter *γ* so as to guarantee *T* ^*γ*^ > *T*_⋆_ for any given *T*_0_ < *T*_⋆_. Denote by *T* = min⁡{*T*_⋆_, *T* ^*γ*^} and by *C* > 0 a constant which depends on *T*_0_.

### 3.1. Derivation of Error Equations and *L* ^2^ Estimate

Let (*u*, *E*, *B*) be the unknown solution to the problem (1) and let (*u* ^*I*^, *p* ^*I*^) be the solution to the incompressible Euler equations (2) defined on [0, *T*_⋆_) given by Proposition 3. Denote this by

(11) $$\begin{array}{c} \left(U,F,G\right)=\left(u-u^{0},E-\nabla p^{I},B-\gamma b^{I}\right), \end{array}$$

which satisfies the following problem:

(12) ∂tU+(U+uI)·∇U=−F−U×(G+γbI)−uI×(G+γbI)−U·∇uI,∂tG−1γ∇×F=−γ∂tbI,U=1γ∇×G, div⁡G=0

with the initial data

(13) $$\begin{array}{c} {\left(U,G\right)|}_{t=0}=\left(u_{0}-u_{0}^{I},B_{0}-\gamma b^{I}\left(t=0\right)\right). \end{array}$$

Based on *L* ^2^-conservation of solutions to the e-MHD system, we obtain *L* ^2^ estimates of the error function (*U*, *F*, *G*). Our basic idea is to cancel the oscillations of the electric field *F* and the magnetic field *G* by using the special structures of the e-MHD system.

**Lemma 5**

For all 0 < *t* < *T* and sufficiently small *γ*, it holds that

(14) ∫Ω(|U|2+|G|2)(t)dx≤∫Ω(|U|2+|G|2)(t=0)dx+C∫0t(||U||2+||G||2)(s)ds+Cγ2.

**Proof**

Taking the *L* ^2^ inner product of the first equation in the error system (12) for *U*, by integration by parts, we get

(15) 12ddt(U,U)=−(F,U)−uI×(G+γbI,U)−(U·∇uI,U)=A1+A2+A3,

where (·, ·) stands for the *L* ^2^ inner product of two scalar or vector functions in *Ω*. Now we estimate each term on the right-hand side of (15).

For *A*_1_, by using the equation *U* = (1/*γ*)∇×*G* in (12) and vector analysis formula

(16) $$\begin{array}{c} \mathrm{div}\left(f\times g\right)=\nabla \times f\cdot g-\nabla \times g\cdot f, \end{array}$$

we have

(17) $$\begin{array}{c} A_{1}=-\frac{1}{\gamma }\left(F,\nabla \times G\right)=-\frac{1}{\gamma }\left(\nabla \times F,G\right). \end{array}$$

For *A*_2_ and *A*_3_, using the property of the approximate solution *u* ^*I*^, Cauchy-Schwarz's inequality, and Minkowski's inequality, we have

(18) $$\begin{array}{c} A_{2}\leq C\left(\gamma +||G||\right)||U||\leq C\gamma^{2}+C\left({||G||}^{2}+{||U||}^{2}\right),\\ A_{3}\leq C{||U||}^{2}. \end{array}$$

Combining (17) with (18), we have

(19) $$\begin{array}{c} \frac{1}{2}\frac{d}{dt}\left(U,U\right)\leq -\frac{1}{\gamma }\left(\nabla \times F,G\right)+C\left({||U||}^{2}+{||G||}^{2}\right)+C\gamma^{2}. \end{array}$$

Multiplying the second equation in the error system (12) by *G*, Cauchy-Schwarz's inequality, we get

(20) $$\begin{array}{c} \frac{1}{2}\frac{d}{dt}\left(G,G\right)\leq \frac{1}{\gamma }\left(\nabla \times F,G\right)+C{||G||}^{2}+C\gamma^{2}. \end{array}$$

It follows from (19) and (20) that

(21) $$\begin{array}{c} \frac{d}{dt}\overset{}{\underset{\Omega }{\int }}\left({\left|U\right|}^{2}+{\left|G\right|}^{2}\right)\left(t\right)dx\leq C\left({||U||}^{2}+{||G||}^{2}\right)+C\gamma^{2}. \end{array}$$

This completes the proof of Lemma 5.

### 3.2. High Order Energy Estimates

We differentiate (12) with ∂_*x*_^*α*^ for a multi-index *α* ∈ *ℕ* ^3^ satisfying |*α*| ≤ *s*_0_ with *s*_0_ > (3/2) + 1 to get that

(22) $$\begin{array}{c} \left(U_{\alpha },F_{\alpha },G_{\alpha }\right)=\left(\partial_{x}^{\alpha }U,\partial_{x}^{\alpha }F,\partial_{x}^{\alpha }G\right) \end{array}$$

satisfies the following problem:

(23) $$\begin{array}{c} \partial_{t}U_{\alpha }+\left(U+u^{I}\right)\cdot \nabla U_{\alpha }=-F_{\alpha }+\overset{4}{\underset{i=1}{\sum }}{ℋ}_{1i},\\ \partial_{t}G_{\alpha }-\frac{1}{\gamma }\nabla \times F_{\alpha }=-\gamma \partial_{t}b_{\alpha }^{I},\\ U_{\alpha }=\frac{1}{\gamma }\nabla \times G_{\alpha },\;\;\mathrm{div}G_{\alpha }=0, \end{array}$$

where

(24) $$\begin{array}{c} {ℋ}_{11}=\left(U+u^{I}\right)\cdot \nabla U_{\alpha }-{\left[\left(U+u^{I}\right)\cdot \nabla U\right]}_{\alpha },\\ {ℋ}_{12}=-{\left[U\times \left(G+\gamma b^{I}\right)\right]}_{\alpha },\\ {ℋ}_{13}=-{\left[u^{I}\times \left(G+\gamma b^{I}\right)\right]}_{\alpha },\\ {ℋ}_{14}=-{\left[U\cdot \nabla u^{I}\right]}_{\alpha }. \end{array}$$

Before performing the energy estimate, we set

(25) $$\begin{array}{c} {ℰ}_{s_{0}}\left(t\right)={||\left(U,G\right)||}_{H^{s_{0}}(\Omega )}. \end{array}$$

**Lemma 6**

For all 0 < *t* < *T* and sufficiently small *γ*, it holds that

(26) ∫Ω(|Uα|2+|Gα|2)(t)dx≤∫Ω(|Uα|2+|Gα|2)(t=0)dx+C∫0t(1+ℰs0(s))ℰs02(s)ds+Cγ2.

**Proof**

Taking the *L* ^2^ inner product of the first equation in (23) with *U*_*α*_, one gets, by using the third equation in (23) and integration by parts, that

(27) 12ddt(Uα,Uα)=−1γ(Fα,∇×G)+∑i=14(ℋ1i,Uα)=−1γ(∇×Fα,Gα)+∑i=14(ℋ1i,Uα).

By using Cauchy-Schwarz's inequality, the Moser-type calculus inequalities in Lemma 1, and Sobolev's lemma, we have that

(28) (ℋ11,Uα)≤C(||∇(U+uI)||L∞||D|α|−1∇U||  +||D|α|(U+uI)||||∇U||Hs0−1)||Uα||≤C(||∇(U+uI)||Hs0−1||D|α|−1∇U||  +||D|α|(U+uI)||||∇U||L∞)||Uα||≤C(1+||U||Hs0)||U||Hs02≤C(1+ℰs0(t))ℰs02(t),(ℋ12,Uα)≤C(1+ℰs0(t))ℰs02(t),(ℋ13,Uα)≤Cℰs02(t)+Cγ2,(ℋ14,Uα)≤Cℰs02(t).

Combining (27) with (28) together, one gets

(29) 12ddt(Uα,Uα)≤−1γ(Fα,∇×Gα)+C(1+ℰs0(t))ℰs02(t)+Cγ2.

Taking the *L* ^2^ inner product of the second equation in (23) with *G*_*α*_, one gets, by using Cauchy-Schwarz's inequality,

(30) 12ddt(Gα,Gα)=1γ(∇×Fα,Gα)−(γ∂tbαI,Gα)≤1γ(∇×Fα,Gα)+C||Gα||2+Cγ2≤1γ(∇×Fα,Gα)+Cℰs02(t)+Cγ2.

Combining (29) and (30), one gets

(31) ddt∫Ω(|Uα|2+|Gα|2)(t)dx≤C(1+ℰs0(t))ℰs02(t)+Cγ2,

which yields (26).

### 3.3. The End of Proof of Theorem 4

Now we let

(32) $$\begin{array}{c} z\left(t\right)={ℰ}_{s_{0}}^{2}\left(t\right). \end{array}$$

Then it follows from Lemmas 5 and 6 that there exists *γ*_0_ > 0, depending only on *T*_0_, such that, for any 0 < *γ* ≤ *γ*_0_ and any 0 < *t* < *T*,

(33) $$\begin{array}{c} z\left(t\right)\leq Cz\left(t=0\right)+C\overset{t}{\underset{0}{\int }}\left(\left(1+\sqrt{z}\right)z\right)\left(s\right)ds+C\gamma^{2}. \end{array}$$

Since *z*(*t* = 0) ≤ *Cγ* ^2^ for some positive constant *C*, now applying Gronwall's inequality to (33), one can conclude that there exists an *γ*_0_ > 0 sufficiently small such that, for any 0 < *γ* ≤ *γ*_0_ and any 0 < *t* < *T*,

(34) $$\begin{array}{c} z\left(t\right)\leq C\gamma^{2}. \end{array}$$

Thus, using the a priori estimate (10), by the extension argument, we can conclude that *T* ^*γ*^ > *T*_0_ for any *T*_0_ < *T*_⋆_.

The proof of Theorem 4 is complete.
